# Higher NLR Values Can Predict Gram-Negative Spontaneous Bacterial Peritonitis and a High In-Hospital Mortality Rate in Patients with Spontaneous Bacterial Peritonitis

**DOI:** 10.3390/life15091363

**Published:** 2025-08-28

**Authors:** Sergiu Marian Cazacu, Ovidiu Mircea Zlatian, Dragos Ovidiu Alexandru, Elena Leocadia Plesea, Ioan Alexandru Vacariu, Mihai Cimpoeru, Ion Rogoveanu, Camelia Cristiana Bigea, Cristina Maria Marginean, Sevastita Iordache

**Affiliations:** 1 Gastroenterology Department, University of Medicine and Pharmacy Craiova, Petru Rares Street no 2-4, 200349 Craiova, Romania; sergiu.cazacu@umfcv.ro (S.M.C.); ion.rogoveanu@umfcv.ro (I.R.); sevastita@gmail.com (S.I.); 2 Microbiology Department, University of Medicine and Pharmacy Craiova, Petru Rares Street no 2-4, 200349 Craiova, Romania; ovidiu.zlatian@umfcv.ro; 3 Statistics Department, University of Medicine and Pharmacy Craiova, Petru Rares Street no 2-4, 200349 Craiova, Romania; dragos.alexandru@umfcv.ro; 4 Doctoral School, University of Medicine and Pharmacy Craiova, Petru Rares Street no 2-4, 200349 Craiova, Romania; alexvacariu@yahoo.com (I.A.V.); camelia_bigea@yahoo.com (C.C.B.); 5 Resident Physician, Emergency Clinic Hospital Craiova, 200642 Craiova, Romania; mcimpoeru810@gmail.com; 6 Internal Medicine Department, University of Medicine and Pharmacy Craiova, Petru Rares Street no 2-4, 200349 Craiova, Romania; marginean22@yahoo.com

**Keywords:** spontaneous bacterial peritonitis, antibiotic susceptibility, Gram-positive bacteria, Gram-negative bacteria

## Abstract

Background: Spontaneous bacterial peritonitis (SBP) represents a significant complication of liver cirrhosis; Gram-positive bacteria (GPB) predominance was recently noted in some areas, with increased antibiotic resistance. Etiology and mortality prediction are important in culture-negative SBP and for empirical antibiotherapy before the arrival of culture results. Methods: A retrospective study was performed on patients with cirrhosis and ascites admitted between 2018 and 2024, divided into culture-positive SBP (Gram-positive and Gram-negative), culture-negative SBP, and non-infected ascites. The NLR (neutrophil-to-lymphocyte ratio) accuracy for the prediction of SBP and in-hospital mortality was estimated using ROC analysis. Results: Overall, 45 culture-positive SBP, 28 culture-negative SBP, and 600 control ascites were diagnosed; Gram-positive SBP represented 60%; median NLR values were significantly higher in patients with Gram-negative compared with Gram-positive SBP (8.79 in Gram-negative versus 3.92 in Gram-positive SBP, AUC 0.752, *p* = 0.003); and a limited role in SBP prediction was recorded (AUC 0.642, *p* = 0.003), with no difference between culture-positive and culture-negative SBP. The NLR median values were higher for patients who died in hospital in all patients with cirrhosis, in SBP, and culture-positive SBP, but not in culture-negative SBP. Conclusions: Higher NLR values were associated with Gram-negative SBP etiology and with in-hospital mortality in all cirrhosis, in SBP, and especially in culture-positive and Gram-negative SBP cases. High NLR values can predict the Gram-negative etiology in patients with an ascitic neutrophil count above 250/mm^3^, which can be used to guide empirical antibiotherapy until cultures are available or in culture-negative SBP.

## 1. Introduction

Bacterial infections are often encountered in liver cirrhosis and are significantly associated with mortality risk [1,2,3,4]; immunosuppressive status, enteric flora abnormalities, bacterial translocation, and intestinal barrier disturbances can explain the high risk of infection [3,4,5]. Spontaneous bacterial peritonitis (SBP) is defined as the appearance of ascitic infection without an intra-abdominal surgically treatable source of infection [6], with a prevalence of 10–30% in hospitalized patients and 20% in-hospital mortality [6,7]. Nosocomial SBP represents cases diagnosed after 48 h of admission, whereas community-acquired SBP are cases diagnosed during the first 48 h after admission without hospital admissions 90 days before the diagnosis; a recently described form, healthcare-associated SBP, is defined by a diagnosis made in the first 24 h after admission in patients previously hospitalized in the last 90 days [8].

Ascitic fluid examination represents the standard diagnostic modality in SBP, with a neutrophil count above 250/mm^3^ being suggestive of the diagnosis [1,6,8]. A total of 40–90% of cultures are positive; some measures, such as bedside inoculation, may increase the percentage of positive cases [1,8]. The main source of SBP causal agents is represented by the gut flora (the most involved bacteria being *E. coli*, *Streptococcus* spp., *Staphylococcus*, *Enterococcus faecalis* and *faecium*, and *Klebsiella* spp.). Gram-negative bacteria are typically involved in SBP [1,8,9,10,11]; however, a microbial shift has been noted lately in many countries, with an increasing frequency of Gram-positive SBP [11,12,13,14]. An increased prevalence of antibiotic-resistant strains was also recorded in SBP, which can complicate the therapy of SBP and can increase mortality [15].

The prediction of Gram-positive or Gram-negative SBP etiology can be important for empirical treatment (before culture results arrive) and in culture-negative SBP. The correct choice of antibiotic is associated with a better chance of survival, and inadequate antibiotherapy may be correlated with a higher risk of death, especially in cases with advanced cirrhosis, significant comorbidities, or severe complications (acute kidney injury, bleeding, or encephalopathy). In culture-negative SBP, empirical treatment cannot be adjusted by antibiogram results; clinical evolution, together with a second examination of ascitic fluid, is currently used to guide therapy.

The neutrophil-to-lymphocyte ratio (NLR) is related to the balance between inflammation and immunoregulatory mechanisms; a prognostic role has been described in acute pancreatitis [16], severe burns [17], sepsis [18], COVID-19 pneumonia [19,20,21,22], encephalopathy [23], and TIPS- and virus C-cirrhosis complications [24,25]. In SBP, several studies have assessed its prognostic role [26,27,28,29,30,31,32,33], and the differences in median NLR value between Gram-negative and Gram-positive infections have been recorded in some studies [34,35,36,37,38,39,40]. The NLR’s predictive role for SBP presence in cirrhosis and for SBP Gram-negative or Gram-positive etiology can help select empirical antibiotherapy until the ascitic neutrophil count and culture are available, given that Gram-positive and Gram-negative bacteria are susceptible to different types of antibiotics.

Our study aimed to assess the predictive role of the NLR in patients with culture-positive and culture-negative SBP versus cirrhosis without SBP, for Gram-negative versus Gram-positive SBP, and for mortality prediction in patients with SBP.

## 2. Materials and Methods

### 2.1. Study Design and Population

**Figure 1:**
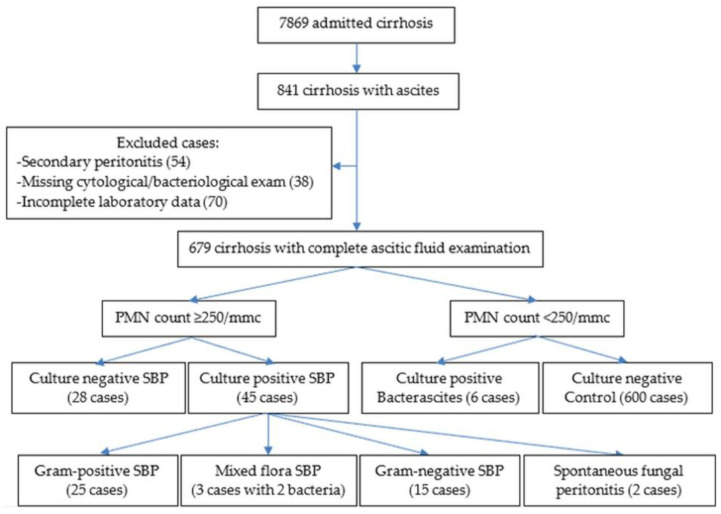
Flowchart for patients enrolled in our study.

A retrospective cohort study during seven years (2018–2024) was conducted; all patients with a diagnostic paracentesis admitted to the Emergency Clinical Hospital Craiova were included. Cases with incomplete data and ascitic fluid infections in patients with recent surgical intervention or peritoneal dialysis (considered as secondary peritonitis) were excluded from the study. The diagnosis of culture-positive SBP was based on an ascitic neutrophil count above 250/mm^3^ and a positive culture from ascitic fluid; patients with negative cultures and an ascitic neutrophil count above 250/mm^3^ were diagnosed as culture-negative SBP; positive ascitic culture cases associated with a neutrophil count less than 250/mm^3^ in ascitic fluid were designated as bacterascitis (Table 1, Figure 1). We divided SBP into non-nosocomial (if paracentesis was performed less than 48 h after admission) and nosocomial (paracentesis performed more than 48 h after admission); in the non-nosocomial group, only one case comprised healthcare-associated SBP. Patients with no ascitic infection and an ascitic neutrophil count below 250/mm^3^ were used as the control group; patients with cirrhosis and other potential causes of ascites (peritoneal tuberculosis, peritoneal carcinomatosis, congestive heart failure, or acute pancreatitis), surgery up to 3 months before paracentesis, and a platelet or blood transfusion before admission were excluded from the control group.

We used the same database as another published study [41]. Collected data were tabulated in an Excel database, and patients with incomplete biological laboratory data were excluded.

The neutrophil-to-lymphocyte ratio (NLR) was calculated at admission (NLR-0) and 48 h (NLR-48). The NLR-0 value was used for the differentiation between SBP and the control group, and between SBP subgroups (culture-positive versus culture-negative SBP, and Gram-positive versus Gram-negative SBP). For prognosis assessment, we analyzed in-hospital mortality, 30-day, 90-day, and 1-year mortality, using stratification risk factors for mortality such as age, Gram-negative or Gram-positive SBP etiology, comorbidities (hepatocellular carcinoma, portal vein thrombosis, cardiovascular diseases, diabetes, gastrointestinal bleeding, and Clostridium difficile infection), biochemical parameters (albumin, bilirubin, creatinine, Na, INR, and hemogram), the presence of acute kidney failure and encephalopathy, Child–Pugh–Turcotte (CTP), and creatinine-CTP, MELD, MELD-3, and MELD-Na scores.

### 2.2. Institutional Protocol Approval

This study was approved by the Institutional Ethics Committee of the Emergency Clinical Hospital of Craiova (Approval Number 10580/3 March 2025).

### 2.3. Outcomes

The primary outcome was the in-hospital mortality rate. The predictive role of NLR-0 for mortality, for SBP presence, and Gram-positive versus Gram-negative etiology was also assessed.

### 2.4. Statistical Analysis

We used Microsoft Excel (Microsoft Corp., Redmond, WA, USA), and the XLSTAT 2016 add-on for MS Excel (Addinsoft SARL, Paris, France) was used for data processing. For categorical variables, the percentages were calculated, while for continuous variables, the median with the interquartile range was estimated. The Kruskal–Wallis test was used for assessing proportion differences between Gram-positive, Gram-negative SBP, and the control group; *p*-values < 0.05 were considered significant. A univariate and multivariate logistic analysis was performed to evaluate the factors associated with both the risk and mortality in Gram-positive and Gram-negative SBP; factors identified through the univariate logistic regression analysis with *p* < 0.2 were included in the multivariate model. The area under the ROC curve (AUC) was constructed, and sensitivity, specificity, and cutoff values were assessed.

## 3. Results

### 3.1. Characteristics of the Patient Group

Overall, 45 patients with spontaneous bacterial or fungal peritonitis were selected over 7 years; 28 culture-negative SBP were also diagnosed during the same period, and 600 patients with cirrhosis and ascites, with complete laboratory data and diagnostic paracentesis, were included as the control group. Age and gender were similar. In all subgroups, most patients had alcoholic cirrhosis and Child B or C class cirrhosis, although in patients with culture-negative SBP, the proportion of patients with alcoholic cirrhosis was lower, and the proportion of patients with viral cirrhosis was higher than in patients with culture-positive SBP and the control group. No differences in pulse or systolic blood pressure were noted between the SBP and control patients. Leucocyte and neutrophil counts were higher in patients with SBP (both culture-positive and culture-negative), and mean urea, creatinine, and total bilirubin levels were also higher; albumin levels were lower. The MELD-3 and MELD-Na levels were also higher in SBP, regardless of the culture status, whereas for the CTP and CTP-creatinine levels, there was no statistically significant difference. Portal vein thrombosis and Clostridium difficile colitis were more frequent in both culture-positive and culture-negative SBP; hepatocellular carcinoma was more frequent in culture-positive SBP than in the control group (*p* = 0.0323). The in-hospital mortality rate was 26.7% for culture-positive SBP/SFP compared with 21.2% for culture-negative SBP (Odds Ratio 1.33, 95%CI 0.44–4.08, *p* = 0.5799), and 6% for cirrhosis without ascitic fluid infection or SBP (Odds Ratio 5.70, 95%CI 2.71–11.96, *p* = 0.6142); the 1-month, 3-month, and 12-month mortality rates were similar in culture-positive and culture-negative SBP and higher than in patients with no ascitic fluid infection (Table 2).

In patients with culture-positive SBP, 10 polymicrobial infections were found (22.2%), 21 infections were non-nosocomial (20 community-acquired and 1 healthcare-associated), and 24 were nosocomial (53.3%). The etiology of culture-positive SBP (Table 3) was dominated by Gram-positive bacteria in both non-nosocomial (59.3%) and nosocomial infections (60.7%); Candida peritonitis was recorded in two cases (3.6%).

From 10 polymicrobial infections, 4 were produced by two Gram-positive bacteria and were included in the Gram-positive subgroup (21 monomicrobial and 4 polymicrobial SBP), 3 were caused by two Gram-negative bacteria and were included in the Gram-negative subgroup (12 monomicrobial and 3 polymicrobial SBP), and the other 3 were produced by an association of one Gram-positive and one Gram-negative bacteria, and were included in the mixed-type SBP.

### 3.2. Predictive Role of NLR in the Type of SBP

**Figure 2:**
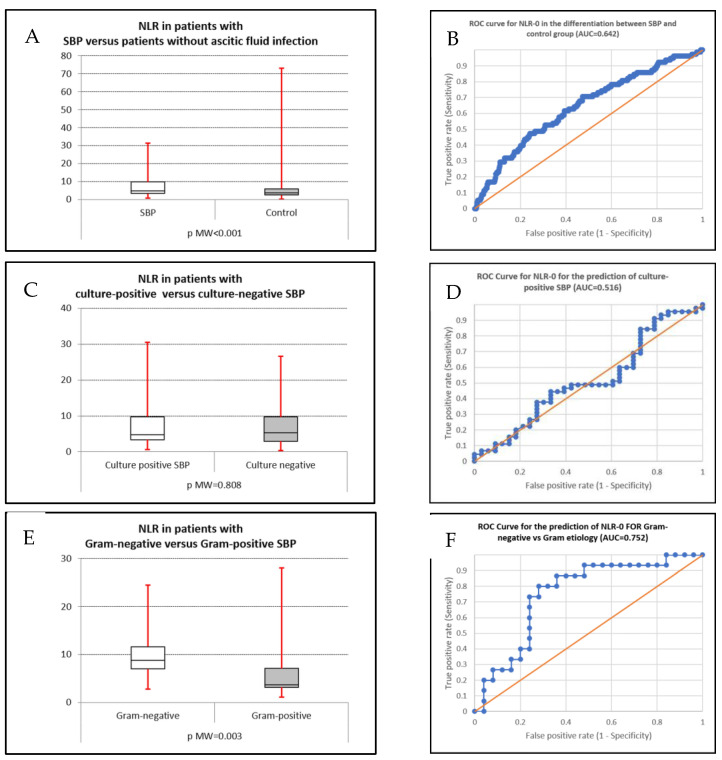
(**A**,**B**) Box plot and AUC for predicting the presence of SBP; (**C**,**D**) box plot and AUC for predicting the presence of culture-positive SBP; (**E**,**F**) box plot and AUC for predicting the presence of Gram-positive or Gram-negative SBP.

We constructed the AUC for NLR-0 for discriminating between SBP patients and the control group, between culture-positive and culture-negative types, and between Gram-positive and Gram-negative SBP (Figure 2A–F).

For SBP presence, an AUC value of 0.642 was recorded, which suggests a mild predictive value with statistical significance (*p* < 0.001). At a cutoff of 6.072, low sensitivity (47.4%) but moderate specificity (75.9%) was noted; at a lower cutoff value of 3.817, moderate sensitivity (70.5%) but low specificity (52.8%) was obtained. For the differentiation between culture-positive SBP and the control group, the obtained AUC was 0.650 (*p* = 0.001); at a cutoff of 4.457, 60% sensitivity and 60.8% specificity were noted. For the differentiation between Gram-positive and Gram-negative SBP, the AUC was 0.752 (*p* = 0.003); at a 6.371 cutoff value, the sensitivity was 80% and the specificity was 72% (Table 4).

### 3.3. Predictive Role of NLR for In-Hospital Mortality

**Figure 3:**
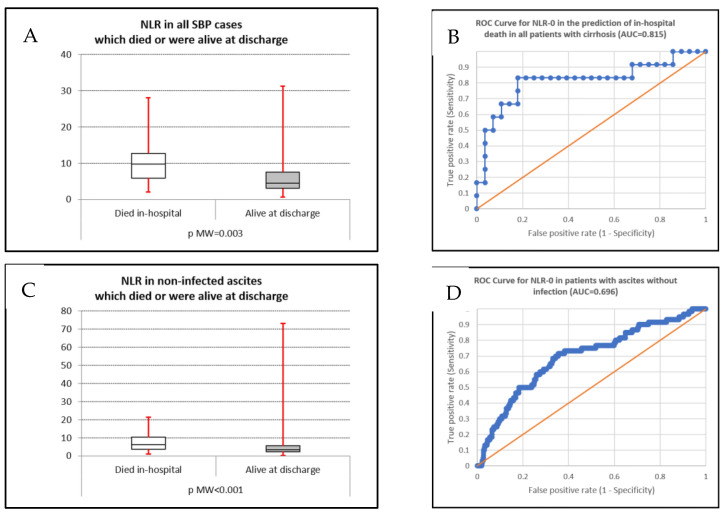
(**A**,**B**) Box plot and AUC for predicting in-hospital mortality in patients with SBP; (**C**,**D**) box plot and AUC for predicting in-hospital mortality in patients with cirrhosis and no ascitic infection.

We constructed the AUC for NLR-0 in predicting in-hospital mortality in all patients with cirrhosis and ascites, SBP subgroups (culture-positive and negative, Gram-positive, and Gram-negative), and patients with non-infected ascites (Figure 3A–D).

**Figure 4:**
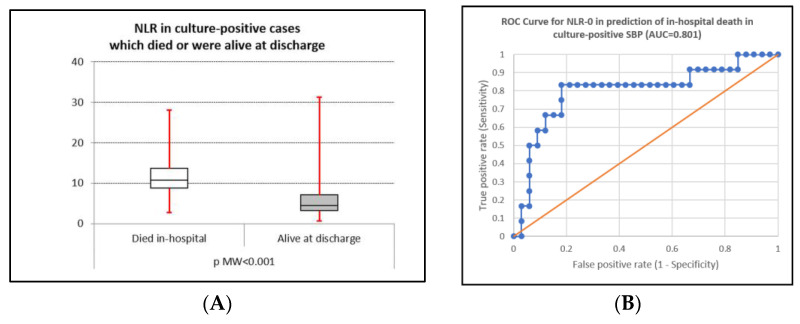

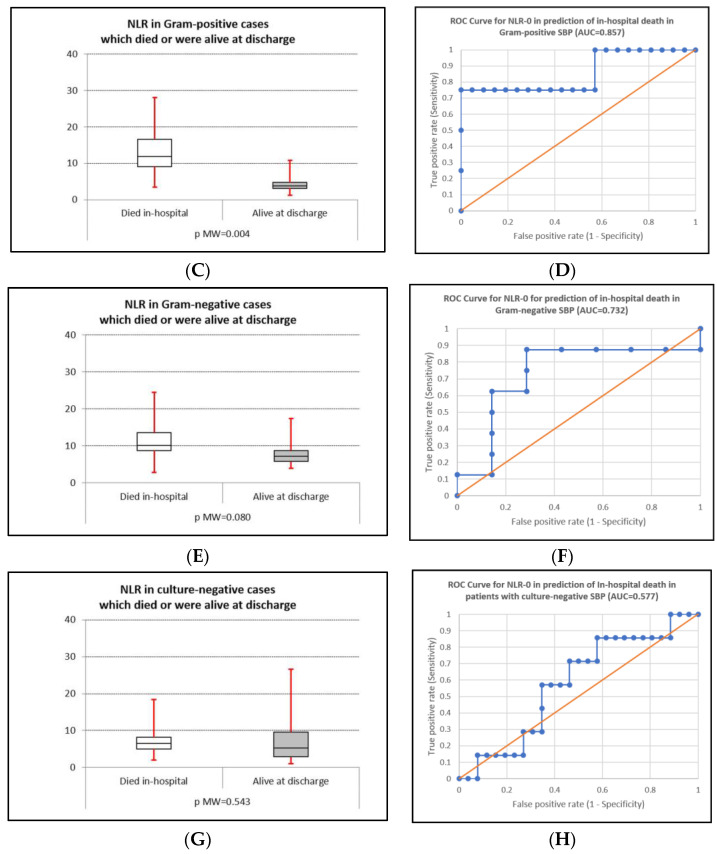
(**A**,**B**) Box plot and AUC for predicting in-hospital mortality in patients with culture-positive SBP; (**C**,**D**) box plot and AUC for predicting in-hospital mortality in patients with Gram-positive SBP; (**E**,**F**) box plot and AUC for predicting in-hospital mortality in patients with Gram-negative SBP; (**G**,**H**) box plot and AUC for predicting in-hospital mortality in patients with culture-negative SBP.

The NLR value had good accuracy for in-hospital mortality prediction in all patients with cirrhosis with ascites, in patients with SBP, in culture-positive SBP, in Gram-positive and Gram-negative SBP, and also in the control group (Figure 4A–H). The highest accuracy provided by the ROC analysis was recorded in patients with Gram-positive SBP (0.857); the lowest statistically significant value was found in the control group (AUC 0.696). In culture-negative SBP, the obtained AUC was 0.577, and statistical significance was not reached (*p* = 0.543)—Table 5.

## 4. Discussion

Infections in patients with liver cirrhosis appear frequently because of immunosuppression status, dysbiosis, and abnormalities in the intestinal barrier, and are associated with a significant mortality risk [3,4,5,42,43]. One of the most severe infections in cirrhosis is represented by spontaneous bacterial/fungal peritonitis. In recent decades, an etiological microbial shift to more frequent Gram-positive SBP coupled with increased resistance to commonly used antibiotics constitutes a significant challenge for management and prognosis. Many studies have shown both increasing Gram-positive etiology and more MDR bacterial infections in liver cirrhosis and SBP [13,15,44,45,46,47,48,49,50,51,52,53,54,55,56,57,58], with geographic variations, which can alter current guidelines regarding SBP therapy and may impose local guidelines adapted to the antibiotic resistance particularities [41]. The etiological spectrum of SBP in our study was characterized by the predominance of Gram-positive bacteria, as in other studies [45,47,48]. A total of 21.6% had polymicrobial peritonitis, and two cases had Candida albicans peritonitis.

The NLR value reflects an imbalance between acute or chronic inflammation and immunoregulatory mechanisms [26,28,29]. In patients with cirrhosis, increased NLR values may be associated with chronic hepatic inflammation and persistent endotoxinemia related to increased gut permeability [26,28]. A more advanced liver disease or acute complications, such as sepsis, SBP, acute infection, encephalopathy, or gastrointestinal bleeding, may be accompanied by higher NLR values, thus emphasizing a prognostic role of the NLR [26,28,31,59]. Some scores, which include age and CRP [33], age, MPV, NLR, and CRP [30,31], or age, gender, NLR, MPV, INR, and total bilirubin [32], have also been suggested for SBP prediction and validated in several studies.

In our study, NLR had limited predictive value for the presence of SBP in patients with liver cirrhosis (AUC 0.642, *p* < 0.001); this finding was discordant to other studies [26,28,60]. A potential explanation may be related to the presence of bacterial translocation and chronic endotoxinemia in patients with liver cirrhosis, even in the absence of a confirmed infection [26]. The predominance of Gram-positive bacteria in patients with SBP in our study may be another explanation, because we found a lower median NLR value in Gram-positive than in Gram-negative SBP. This finding is highlighted by the higher predictive value of NLR in Gram-negative SBP compared with the control group (AUC 0.814, *p* < 0.001), with both a sensitivity and specificity of 80% for a cutoff value of 6.741. A more frequent presence of other infection types in patients with cirrhosis, especially urinary infections, bloodstream infections, or pneumonia, can be another factor explaining the lack of predictive value of NLR for SBP. However, in our study, we rarely noted both the presence of bloodstream infection (2.2–3.6%) and other types of infection. Contrary to some studies that found higher NLR values in patients with culture-positive SBP [27], we found no significant predictive value for NLR in the differentiation between culture-positive and culture-negative SBP. The lack of NLR accuracy in discriminating between culture-negative and culture-positive SBP can be explained by the fact that culture-positive and culture-negative SBP may be similar in terms of systemic inflammation severity.

An important finding of our study was the potential role of NLR for the differentiation between Gram-negative and Gram-positive SBP, with a median NLR value of 8.79 in Gram-negative and 3.92 in Gram-positive SBP (AUC 0.752, *p* = 0.003). The sensitivity and specificity of the NLR value in discriminating between Gram-negative and Gram-positive SBP were 80% and 72%, respectively, at a cutoff value of 6.371; although not perfect, the NLR value can be of clinical utility in such settings. The distinction between Gram-positive and Gram-negative SBP may be important for two reasons: in patients with an ascitic neutrophil count above 250/mm^3^ (when empirical treatment before a culture with antibiogram arrives may be guided by the presumed Gram-positive or Gram-negative etiology) and in culture-negative SBP (when a high NLR value may suggest a Gram-negative etiology and low NLR values suggests a Gram-positive cause of SBP), thus orienting antibiotherapy until a follow-up paracentesis can be used to adjust the treatment. However, the differences between the median NLR values in Gram-positive and Gram-negative SBP may be partially explained by the higher mortality rate in our Gram-negative SBP, which may impact the accurate differentiation of Gram-positive versus Gram-negative SBP.

The predictive role of the NLR level in assessing the presence, etiology, and outcome of SBP may be explained by multiple factors [34,35,36,37,38,39,40]. An increased count of blood neutrophils and decreased blood lymphocytes can be encountered in severe inflammation [31], and especially in the presence of infection; however, an increased level of proinflammatory and neutrophilic biomarkers was recorded in cirrhotic plasma even without infection (because of endotoxinemia, bacterial translocation, and other factors), together with lymphopenia secondary to hypersplenism. This may explain a reduced role of the NLR level in predicting SBP presence. The association between the NLR and severe inflammation may suggest NLR’s value as a risk factor for poor clinical prognosis [34,37]; inflammation-related stress can induce a redistribution of lymphocytes from the blood to lymphoid tissues, which decreases blood lymphocyte count [34]. The immune host response may vary between Gram-positive and Gram-negative species [38,40], as a result of specific cytokine profiles and contents related to the unique structures and antigenic components [38].

The literature data regarding the NLR’s role in the differentiation and prognosis of Gram-positive and Gram-negative SBP is scarce and somewhat contradictory [34,35,36,37,38,39,40]. Higher NLR values were associated with dismal prognosis in patients with peritoneal dialysis and peritonitis, and Gram-positive spontaneous peritonitis (except for Staphylococcus peritonitis) had a better prognosis than Gram-negative cases [34]. Two studies have shown higher NLR levels in Gram-positive sepsis and septic shock [35,37], having been explained by a stronger activation of NK cells coupled with a slower normalization of T cell lymphocytes and a stronger lymphocyte suppression, a thicker and tighter peptidoglycan layer, a higher IL-12 level, and a stronger CMH class II-response in Gram-positive infections [37]. However, two other studies that include patients with bloodstream infections have shown higher NLR median values in Gram-negative infections, with median values of 12.07 versus 5.68 and 8.25 cutoff [38], and 14.15 versus 9.46 [40], explained by the lipopolysaccharide wall constituent (endotoxin) in Gram-negative bacteria, which increases the neutrophil count and decreases the lymphocyte count [38]. In another study including 58 patients with hemodialysis and catheter-related blood infections, the NLR values were not significantly different between Gram-positive and Gram-negative [36].

The in-hospital mortality rate for culture-positive SBP was 26.7%, similar to culture-negative SBP and data from the literature [1,2,12,42], which reported values of 40% for Gram-negative SBP and 20% for Gram-positive SBP. The mortality seems similar for culture-positive and culture-negative SBP in both short-term (in-hospital and 30-day) and long-term settings (90-day and 1-year), with higher mortality in both short and long-term settings compared with control ascites. The NLR had significant predictive value in almost all patient groups, except for culture-negative SBP (all cirrhosis with ascites, all SBP, culture-positive SBP, Gram-positive SBP, Gram-negative SBP, and control group), with the highest accuracy in Gram-positive SBP (AUC 0.857) and the lowest accuracy in the control group (AUC 0.696). This finding is similar to a study where a mean NLR level of 16.5 ± 11.8 was noted in patients who died during admission compared with 7.8 ± 9 in those who survived [26]. For patients with culture-negative SBP, we may presume that the absence of an isolated strain from the ascitic fluid may be explained by a lower colonization of the ascitic fluid and, subsequently, a lower systemic inflammatory response, thus obscuring the differences between culture-negative SBP and the control group. Supplementary studies, including other biological inflammatory markers such as (but not limited to) C-reactive protein, IL-8, and other markers, may help to elucidate such a finding.

Although liver cirrhosis is an inflammatory state as a result of a leaky gut, bacterial translocation from the bowel, and the presence of dysbiosis and proinflammatory cytokines released by hepatocyte necrosis, other factors can also influence the inflammatory balance in patients with cirrhosis. The presence of other infections outside SBP may also increase NLR levels, especially in the presence of bacteriemia [35,39]. Malnutrition is frequently noted in patients with advanced liver disease, and an NLR above 4.2 was associated with a higher malnutrition score, with NLR being a risk factor for malnutrition independently of alcoholic etiology and ascite presence [61]. The NLR level in patients with hepatocellular carcinoma was associated with overall and disease-free survival, with higher NLR values in patients with poor prognosis, and lower values associated with better overall, recurrence-free, and disease-free survival [62,63]. In our study, positive blood cultures were rarely present in all groups (2.2–3.6%). The nutritional status could not be adequately assessed due to the retrospective design and insufficient data for the accurate estimation of dietary scores. No significant differences regarding the proportion of HCC were recorded between patients with culture-positive and culture-negative SBP, and the control group (Chi-square *p*-value = 0.072). Seven cases of hepatocellular carcinoma were noted in the culture-positive SBP group, four with Gram-positive, three with Gram-negative SBP, and none with in-hospital mortality. The mean NLR was 8.54 ± 0.8 for patients with culture-positive SBP and hepatocellular carcinoma and 7.26 ± 0.4 for patients without hepatocarcinoma (*p*-value Kruskal–Wallis 0.260).

Our study had some limitations. The small number of patients in our study was associated with the monocentric nature, which may limit the generalizability of the obtained findings; this is particularly true for smaller subgroup analyses, such as Gram-positive or Gram-negative SBP. The retrospective design made other potentially inflammatory biomarkers, such as TNF-alpha, IL-6, MIP-1β, lactoferrin, procalcitonin, or calprotectin, unavailable for a complementary role, especially in predicting Gram-negative or Gram-positive etiology. A multicentric study or an extended study timeframe may overcome some of the limitations and increase data accuracy.

## 5. Conclusions

A high NLR value at admission was associated with moderate but significant predictive value for Gram-negative spontaneous bacterial peritonitis, and moderate predictive value for in-hospital mortality in patients with cirrhosis and ascites with and without SBP (except for those with culture-negative SBP). The predictive value in differentiating between Gram-positive and Gram-negative SBP, eventually in association with other markers of systemic inflammation, may aid in the antibiotherapy choice in patients with a high ascitic neutrophil count before culture results with antibiogram or in those with culture-negative spontaneous bacterial peritonitis. However, given the paucity of data regarding the potential role in predicting Gram-positive versus Gram-negative etiology, supplementary studies and corroboration with other systemic inflammation markers are necessary to assess the exact role of NLR in predicting the type of infection.

## Figures and Tables

**Table 1:** Definition criteria for culture-positive, culture-negative SBP, and control.

SBP	Ascitic PMN count ≥ 250/mm^3^
Culture-positive SBP	Ascitic PMN count ≥ 250/mm^3^ + a positive ascitic culture
Culture-negative SBP	Ascitic PMN count ≥ 250/mm^3^ + a negative ascitic culture
Bacterascitis	Ascitic PMN count < 250/mm^3^ + a positive ascitic culture
Control Group	Ascitic PMN count < 250/mm^3^ + a negative ascitic culture

**Table 2:** Characteristics of patients with culture-positive and culture-negative SBP/SFP, and control.

	Culture-Positive SBP (N = 45)	Culture-Negative SBP (N = 28)	Control (N = 600)	*p*-Value
Age (years); mean± std dev (range)	60.1 ± 12.1	59.4 ± 11.4	59.2 ± 11.3	0.883
Gender Males/Females (% Males)	29/16 (64.4)	22/6 (78.6)	392/208 (65.3)	0.346
Cirrhosis etiology (%)				
Alcoholic	36 (80)	15 (53.6)	474 (79.0)	0.017
Hepatitis B or/and C	6 (13.3)	10 (35.7)	69 11.5)	
Mixed (viral + alcoholic)	3 (6.7)	3 (10.7)	53 (8.8)	
Other (autoimmune, biliary)	0 (0)	0 (0)	4 (0.7)	
Systolic blood pressure (mean ± stdev)	117.9 ± 18.7	115.0 ± 22.5	121.9 ± 20.8	0.272
Pulse (count/min, mean ± stdev)	89.7 ± 18.8	91.6 ± 19.4	87.5 ± 17.9	0.343
Laboratory analyses (mean ± stdev)				
Hemoglobin (g/dL)	9.98 ± 2.29	10.56 ± 2.25	10.39 ± 2.37	0.555
Leucocyte (count/mm^3^)	10,991 ± 6611	11,190 ± 5378	8713 ± 5182	0.000
Neutrophil (count/mm^3^)	8371 ± 6138	8082 ± 3960	6065 ± 4470	<0.0001
Lymphocyte (count/mm^3^)	1416 ± 818	1861 ± 2487	1564 ± 989	0.670
Platelet count (×1000/mm^3^)	172,563 ± 116,111	158,860 ± 103,637	149,639 ± 82,947	0.619
Urea (mg/dL)	56.1 ± 43.9	74.0 ± 55.5	53.0 ± 45.8	0.036
Creatinine (mg/dL)	1.43 ± 2.73	1.61 ± 1.75	1.15 ± 1.31	0.050
INR	1.763 ± 0.503	1.672 ± 0.357	1.643 ± 0.639	0.062
Albumin (g/dL)	2.46 ± 0.60	2.41 ± 0.46	2.63 ± 0.56	0.033
Total bilirubin (mg/dL)	6.16 ± 6.16	7.03 ± 8.46	4.01 ± 4.69	0.018
ALT (UI/dL)	49.9 ± 95.4	38.8 ± 30.4	36.0 ± 34.7	0.341
AST (UI/dL)	119.8 ± 156.1	90.4 ± 80.7	85.0 ± 74.0	0.297
Na (mmol/L)	131.2 ± 6.3	131.7 ± 5.7	132.0 ± 5.9	0.598
K (mmol/L)	4.57 ± 0.88	4.38 ± 0.77	4.36 ± 0.80	0.233
ESR (mm)	57 ± 35	51 ± 27	51 ± 30	0.590
Blood culture positive (%)	1 (2.2)	1 (3.6)	15 (2.5)	0.931
Previous antibiotherapy (%)	21 (46.7)	12 (42.9)	256 (42.7)	0.872
Child class (%) A	1 (2.2)	0 (0)	23 (3.8)	0.562
B	17 (37.8)	10 (35.7)	260 (43.3)	
C	27 (60)	18 (64.3)	317 (52.8)	
Child/MELD score (Mean ± Stdev)				
CTP	9.867 ± 1.804	10.179 ± 1.701	9.703 ± 2.020	0.302
CTP-creatinine	10.578 ± 2.360	11.393 ± 2.558	10.377 ± 2.298	0.109
MELD-3	39.556 ± 23.812	35.857 ± 17.264	29.110 ± 12.378	0.000
MELD-Na	39.044 ± 19.862	36.214 ± 16.008	29.997 ± 11.400	0.000
Complications (%)				
Encephalopathy	14 (31.1)	8 (28.6)	198 (33.0)	0.864
Acute kidney injury				
Comorbidities (%)				
Hepatocellular carcinoma	7 (15.6)	3 (10.7)	40 (6.7)	0.072
Portal vein thrombosis	7 (15.6)	4 (14.3)	36 (6.0)	0.016
Acute variceal bleeding	9 (20)	4 (14.3)	72 (12.0)	0.286
Pneumonia	7 (9.7)	3 (10.7)	58 (15.6)	0.447
Clostridium difficile colitis	3 (10.7)	3 (10.7)	10 (1.7)	0.001
Cardiovascular	4 (19.0)	5 (17.9)	114 (8.9)	0.238
Diabetes	5 (14.0)	4 (14.3)	84 11.1)	0.861
Previous chronic kidney disease	5 (8.5)	3 10.7)	51 (11.1)	0.781
Mortality % in-hospital	26.7	21.4	6.0	<0.0001
30-day	44.4	43.5	13.3	<0.0001
90-day	46.7	54.5	21.2	<0.0001
1-year	73.0	76.2	37.3	<0.0001

INR = International Normalized Ratio, ALT = Alanine Aminotransferase, AST = Aspartate Aminotransferase, CTP = Child–Turcotte–Pugh score, MELD = Model for End-stage Liver Diseases, SBP = Spontaneous Bacterial Peritonitis, SFP = Spontaneous Fungal Peritonitis.

**Table 3:** Etiology for non-nosocomial and nosocomial-associated infection.

	Non-Nosocomial Infection (%)	Nosocomial Infection (%)
G-positive	16 (59.3)	17 (60.7)
*Staphylococcus aureus*	11 (40.7)	12 (41.8)
*Streptococcus* spp.	2 (7.4)	1 (3.6)
*Enterococcus* spp.	2 (7.4)	4 (14.3)
*Staphylococcus coagulase-negative*	1 (3.7)	0 (0)
G-negative	9 (33.3)	11 (39.3)
*E. coli*	3 (11.1)	3 (10.7)
*Klebsiella* spp.	2 (7.4)	3 (10.7)
*Acinetobacter baumanii*	1 (3.7)	3 (10.7)
*Pseudomonas aeruginosa*	1 (3.7)	1 (3.6)
*Citrobacter*	1 (3.7)	0 (0)
*Proteus mirabilis*	1 (3.7)	0 (0)
*Enterobacter* spp.	0 (0)	1 (3.6)
Candida	2 (7.4)	0 (0)
TOTAL	27	28

**Table 4:** NLR-0 median, IQR, cutoff, sensitivity, and specificity for the SBP and control group.

Patient Group	Median (IQR)	AUC	*p*-Value	Cutoff	Sensitivity	Specificity
All SBP	5.30 (3.35–9.81)	0.642	<0.001	3.817	70.5	52.8
Control	3.66 (2.49–5.94)					
Culture-positive SBP	4.75 (3.36–9.77)	0.516	0.808	2.292	91.1	21.2
Culture-negative SBP	5.33 (2.96–9.73)					
Gram-negative SBP	8.79 (7.01–11.61)	0.752	0.003	6.371	80	72
Gram-positive SBP	3.92 (3.20–7.07)					
Gram-negative SBP	8.79 (7.01–11.61)	0.814	<0.001	6.741	80	79.9
Control	3.66 (2.49–5.94)					

IQR = Interquartile range, NLR = neutrophil-to-lymphocyte ratio, SBP = spontaneous bacterial peritonitis.

**Table 5:** Median, IQR, cutoff, sensitivity, and specificity for NLR-0 in patients who died/were alive at discharge.

Patient Group		Median (IQR)	AUC	*p*-Value	Cutoff	Sensitivity	Specificity
All ascites	Dead	7.09 (4.42–11.23)	0.815	<0.001	7.750	83.3	82.1
	Alive	3.63 (2.45–5.80)					
All SBP	Dead	9.73 (5.92–12.75)	0.717	0.003	6.371	73.7	67.8
	Alive	4.54 (3.09–7.61)					
Control	Dead	6.22 (3.71–10.30)	0.696	<0.001	4.524	71.7	64.5
	Alive	3.56 (2.37–5.59)					
Culture+ SBP	Dead	10.70 (8.75–13.6)	0.818	<0.001	7.750	81.8	80.1
	Alive	4.47 (3.20–7.07)					
Culture− SBP	Dead	6.50 (5.00–8.13)	0.577	0.543	4.297	85.7	42.3
	Alive	5.26 (2.90–9.65)					
Gram+ SBP	Dead	11.85 (9.05–16.6)	0.857	0.004	10.821	75.0	100
	Alive	3.82 (3.16–4.74)					
Gram− SBP	Dead	10.13 (8.75–13.6)	0.732	0.080	7.750	87.5	71.4
	Alive	7.22 (5.72–8.69)					

IQR = Interquartile range, NLR = neutrophil-to-lymphocyte ratio, SBP = spontaneous bacterial peritonitis, Culture+ = Culture-positive, Culture− = Culture-negative.

## Data Availability

The data presented in this study are available on request from the corresponding author.

## References

[1] European Association for the Study of the Liver (2018). EASL Clinical Practice Guidelines for the management of patients with decompensated cirrhosis. J. Hepatol..

[2] Bunchorntavakul C., Chamroonkul N., Chavalitdhamrong D. (2016). Bacterial infections in cirrhosis: A critical review and practical guidance. World J. Hepatol..

[3] Ascione T., Di Flumeri G., Boccia G., De Caro F. (2017). Infections in patients affected by liver cirrhosis: An update. Infez. Med..

[4] Piano S., Singh V., Caraceni P., Maiwall R., Alessandria C., Fernandez J., Soares E.C., Kim D.J., Kim S.E., Marino M. (2019). Epidemiology and Effects of Bacterial Infections in Patients with Cirrhosis Worldwide. Gastroenterology.

[5] Voicu M.N., Popescu F., Florescu D.N., Rogoveanu I., Turcu-Stiolica A., Gheonea D.I., Iovanescu V.F., Iordache S., Cazacu S.M., Ungureanu B.S. (2021). Clostridioides difficile Infection among Cirrhotic Patients with Variceal Bleeding. Antibiotics.

[6] Aithal G.P., Palaniyappan N., China L., Härmälä S., Macken L., Ryan J.M., Wilkes E.A., Moore K., Leithead J.A., Hayes P.C. (2021). Guidelines on the management of ascites in cirrhosis. Gut.

[7] Ekpanyapong S., Reddy K.R. (2019). Infections in Cirrhosis. Curr. Treat. Options Gastroenterol..

[8] Biggins S.W., Angeli P., Garcia-Tsao G., Ginès P., Ling S.C., Nadim M.K., Wong F., Kim W.R. (2021). Diagnosis, Evaluation, and Management of Ascites, Spontaneous Bacterial Peritonitis and Hepatorenal Syndrome: 2021 Practice Guidance by the American Association for the Study of Liver Diseases. Hepatology.

[9] Căruntu F.A., Benea L. (2006). Spontaneous bacterial peritonitis: Pathogenesis, diagnosis, treatment. J. Gastrointest. Liver Dis..

[10] Long B., Gottlieb M. (2023). Emergency medicine updates: Spontaneous bacterial peritonitis. Am. J. Emerg. Med..

[11] Fiore M., Di Franco S., Alfieri A., Passavanti M.B., Pace M.C., Kelly M.E., Damiani G., Leone S. (2019). Spontaneous bacterial peritonitis caused by Gram-negative bacteria: An update of epidemiology and antimicrobial treatments. Expert. Rev. Gastroenterol. Hepatol..

[12] Shizuma T. (2018). Spontaneous bacterial and fungal peritonitis in patients with liver cirrhosis: A literature review. World J. Hepatol..

[13] Fiore M., Maraolo A.E., Gentile I., Borgia G., Leone S., Sansone P., Passavanti M.B., Aurilio C., Pace M.C. (2017). Current concepts and future strategies in the antimicrobial therapy of emerging Gram-positive spontaneous bacterial peritonitis. World J. Hepatol..

[14] Nookala V.K., Sharzehi K., Lee T.P. (2012). An Increase of Gram-Positive Bacteria in Spontaneous Bacterial Peritonitis. Gastroenterology.

[15] Oliveira J.C., Carrera E., Petry R.C., Deutschendorf C., Mantovani A., Barcelos S.T.A., Cassales S., Schacher F.C., Lopes A.B., Alvares-da-Silva M.R. (2019). High Prevalence of Multidrug-Resistant Bacteria in Cirrhotic Patients with Spontaneous Bacterial Peritonitis: Is It Time to Change the Standard Antimicrobial Approach?. Can. J. Gastroenterol. Hepatol..

[16] Cazacu S.M., Parscoveanu M., Cartu D., Moraru E., Rogoveanu I., Ungureanu B.S., Iordache S., Florescu D.N., Iovanescu V.F., Dragomir M.I. (2023). NLR48 is Better Than CRP, and mCTSI, and Similar to BISAP and SOFA Scores for Mortality Prediction in Acute Pancreatitis: A Comparison of 6 Scores. J. Inflamm. Res..

[17] Qiu L., Jin X., Wang J.J., Tang X.D., Fang X., Li S.J., Wang F., Chen X.L. (2021). Plasma Neutrophil-to-Lymphocyte Ratio on the Third Day Postburn is Associated with 90-Day Mortality Among Patients with Burns Over 30% of Total Body Surface Area in Two Chinese Burns Centers. J. Inflamm. Res..

[18] Wu H., Cao T., Ji T., Luo Y., Huang J., Ma K. (2024). Predictive value of the neutrophil-to-lymphocyte ratio in the prognosis and risk of death for adult sepsis patients: A meta-analysis. Front. Immunol..

[19] Kosidło J.W., Wolszczak-Biedrzycka B., Matowicka-Karna J., Dymicka-Piekarska V., Dorf J. (2023). Clinical Significance and Diagnostic Utility of NLR, LMR, PLR and SII in the Course of COVID-19: A Literature Review. J. Inflamm. Res..

[20] Ulloque-Badaracco J.R., Ivan Salas-Tello W., Al-Kassab-Córdova A., Alarcón-Braga E.A., Benites-Zapata V.A., Maguiña J.L., Hernandez A.V. (2021). Prognostic value of neutrophil-to-lymphocyte ratio in COVID-19 patients: A systematic review and meta-analysis. Int. J. Clin. Pract..

[21] Turgunova L., Mekhantseva I., Akhmaltdinova L., Kostinov M., Zhumadilova Z., Turmukhambetova A. (2023). Association of sTREM-1 and Neutrophil-to-Lymphocyte Ratio as Prognostic Markers in COVID-19 Short- and Medium-term Mortality. J. Inflamm. Res..

[22] Sarkar S., Khanna P., Singh A.K. (2022). The Impact of Neutrophil-Lymphocyte Count Ratio in COVID-19: A Systematic Review and Meta-Analysis. J. Intensive Care Med..

[23] Zhang L., Zhang W., Wang J., Jin Q., Ma D., Huang R. (2023). Neutrophil-to-lymphocyte ratio predicts 30-, 90-, and 180-day readmissions of patients with hepatic encephalopathy. Front. Med..

[24] Hong C.M., Su T.H., Hsu S.J., Tseng T.C., Liu C.H., Yang H.C., Kao J.H., Chen P.J., Cheng P.N., Hung C.H. (2024). Addition of neutrophil-to-lymphocyte ratio to Pre-DAA FIB-4 does not increase prediction value for de novo liver complications in hepatitis C. J. Formos. Med. Assoc..

[25] Tang H.H., Zhou L.F., Wang C.X., Zha Y., Fan C., Zhong B.Y., Zhu X.L., Wang W.D. (2024). The Value of Neutrophil-to-Lymphocyte Ratio in Predicting Mortality After Transjugular Intrahepatic Portosystemic Shunt Placement. J. Inflamm. Res..

[26] Seyedi S.A., Nabipoorashrafi S.A., Hernandez J., Nguyen A., Lucke-Wold B., Nourigheimasi S., Khanzadeh S. (2022). Neutrophil to Lymphocyte Ratio and Spontaneous Bacterial Peritonitis among Cirrhotic Patients: A Systematic Review and Meta-analysis. Can. J. Gastroenterol. Hepatol..

[27] Efgan M.G., Acar H., Kanter E., Kırık S., Duman Şahan T. (2024). Role of Systemic Immune Inflammation Index, Systemic Immune Response Index, Neutrophil Lymphocyte Ratio and Platelet Lymphocyte Ratio in Predicting Peritoneal Culture Positivity and Prognosis in Cases of Spontaneous Bacterial Peritonitis Admitted to the Emergency Department. Medicina.

[28] Baweja A., Jhamb R., Kumar R., Garg S., Gogoi P. (2021). Clinical utility of Neutrophil Lymphocyte Ratio (NLR) as a marker of Spontaneous Bacterial Peritonitis (SBP) in patients with cirrhosis—An exploratory study. Int. J. Sci. Res. Arch..

[29] Awad S., Ahmed E., Mohamed E. (2020). Role of Combined Blood Neutrophil- Lymphocyte Ratio and C-reactive Protein in Diagnosis of Spontaneous Bacterial Peritonitis. Benha J. Appl. Sci..

[30] Huynh N.C., Vo T.D. (2023). Validation of a new simple scoring system to predict spontaneous bacterial peritonitis in patients with cirrhosis and ascites. BMC Gastroenterol..

[31] Abdel-Razik A.M.N., Abdel-Aziz M., Elsherbiny W., Zakaria S., Shabana W., Abed S., Elhelaly R., Elzehery R., Eldars W., Elbendary M. (2019). Mansoura simple scoring system for prediction of spontaneous bacterial peritonitis: Lesson learnt. Eur. J. Gastroenterol. Hepatol..

[32] Abdo G., Nir U., Rawajdey R., Abu Dahoud W., Massalha J., Hajouj T., Assadi M.H., William N. (2023). A Novel Score-Based Approach by Using Routine Laboratory Tests for Accurate Diagnosis of Spontaneous Bacterial Peritonitis (SBP) in Cirrhotic Patients. EJIFCC.

[33] Mousa N., Besheer T., Abdel-Razik A., Hamed M., Deiab A.G., Sheta T., Eldars W. (2018). Can combined blood neutrophil to lymphocyte ratio and C-reactive protein be used for diagnosis of spontaneous bacterial peritonitis?. Br. J. Biomed. Sci..

[34] Zhou D., Yang H., Zeng L., Yang W., Guo F., Cui W., Chen C., Zhao J., Wu S., Yang N. (2023). Calculated inflammatory markers derived from complete blood count results, along with routine laboratory and clinical data, predict treatment failure of acute peritonitis in chronic peritoneal dialysis patients. Ren. Fail..

[35] Jonathan J., Pradian E., Zulfariansyah A. (2019). Correlation Between Neutrophil-lymphocyte Count Ratio and Procalcitonin in Sepsis and Septic Shock. Maj. Kedokt. Bdg..

[36] Yang J., Wang H., Hua Q., Wu J., Wang Y. (2022). Diagnostic Value of Systemic Inflammatory Response Index for Catheter-Related Bloodstream Infection in Patients Undergoing Haemodialysis. J. Immunol. Res..

[37] Liang P., Yu F. (2022). Predictive Value of Procalcitonin and Neutrophil-to-Lymphocyte Ratio Variations for Bloodstream Infection with Septic Shock. Med. Sci. Monit..

[38] Li X., Yuan X., Wang C. (2019). The clinical value of IL-3, IL-4, IL-12p70, IL17A, IFN-γ, MIP-1β, NLR, P-selectin, and TNF-α in differentiating bloodstream infections caused by gram-negative, gram-positive bacteria and fungi in hospitalized patients: An Observational Study. Medicine.

[39] Roldgaard M., Benfield T., Tingsgård S. (2024). Blood neutrophil to lymphocyte ratio is associated with 90-day mortality and 60-day readmission in Gram negative bacteremia: A multi-center cohort study. BMC Infect. Dis..

[40] Tang W., Zhang W., Li X., Cheng J., Liu Z., Zhou Q., Guan S. (2020). Hematological parameters in patients with bloodstream infection: A retrospective observational study. J. Infect. Dev. Ctries..

[41] Cazacu S.M., Zlatian O.M., Plesea E.L., Vacariu A.I., Cimpoeru M., Rogoveanu I., Bigea C.C., Iordache S. (2025). Predominant Gram-Positive Etiology May Be Associated with a Lower Mortality Rate but with Higher Antibiotic Resistance in Spontaneous Bacterial Peritonitis: A 7-Year Study in a Tertiary Center in Romania. Life.

[42] Tay P.W.L., Xiao J., Tan D.J.H., Ng C., Lye Y.N., Lim W.H., Teo V.X.Y., Heng R.R.Y., Yeow M.W.X., Lum L.H.W. (2021). An Epidemiological Meta-Analysis on the Worldwide Prevalence, Resistance, and Outcomes of Spontaneous Bacterial Peritonitis in Cirrhosis. Front. Med..

[43] Meyyur Aravamudan V., Khan S.R., Hussain I. (2019). Clostridium difficile Infection in Liver Cirrhosis Carries a Higher Risk of Mortality: A Comprehensive Literature Review. Cureus.

[44] de Mattos A.A., Costabeber A.M., Lionço L.C., Tovo C.V. (2014). Multi-resistant bacteria in spontaneous bacterial peritonitis: A new step in management?. World J. Gastroenterol..

[45] Piroth L., Pechinot A., Di Martino V., Hansmann Y., Putot A., Patry I., Hadou T., Jaulhac B., Chirouze C., Rabaud C. (2014). Evolving epidemiology and antimicrobial resistance in spontaneous bacterial peritonitis: A two-year observational study. BMC Infect. Dis..

[46] Pimentel R., Leitão J., Gregório C., Santos L., Carvalho A., Figueiredo P. (2021). Spontaneous Bacterial Peritonitis in Cirrhotic Patients: A Shift in the Microbial Pattern? A Retrospective Analysis. GE-Port. J. Gastroenterol..

[47] Ratnasekera I.U., Johnson A., Powell E.E., Henderson A., Irvine K.M., Valery P.C. (2022). Epidemiology of ascites fluid infections in patients with cirrhosis in Queensland, Australia from 2008 to 2017: A population-based study. Medicine.

[48] Alexopoulou A., Vasilieva L., Agiasotelli D., Siranidi K., Pouriki S., Tsiriga A., Toutouza M., Dourakis S.P. (2016). Extensively drug-resistant bacteria are an independent predictive factor of mortality in 130 patients with spontaneous bacterial peritonitis or spontaneous bacteremia. World J. Gastroenterol..

[49] Khoury A., Rattanasuwan T., Ebied A.M. (2020). Shifting microorganism incidence in cirrhotic patients with ascites: A 5-year retrospective cross-sectional analysis. Dig. Med. Res..

[50] Friedrich K., Nüssle S., Rehlen T., Stremmel W., Mischnik A., Eisenbach C. (2016). Microbiology and resistance in first episodes of spontaneous bacterial peritonitis: Implications for management and prognosis. J. Gastroenterol. Hepatol..

[51] Santoiemma P.P., Dakwar O., Angarone M.P. (2020). A retrospective analysis of cases of Spontaneous Bacterial Peritonitis in cirrhosis patients. PLoS ONE.

[52] Quickert S., Würstle S., Reuken P.A., Hagel S., Schneider J., Schmid R.M., Neugebauer S., Stallmach A., Bruns T. (2022). Real-World Effectiveness of Piperacillin/Tazobactam with and without Linezolid for Spontaneous Bacterial Peritonitis. Dig. Dis..

[53] Shi L., Wu D., Wei L., Liu S., Zhao P., Tu B., Xie Y., Liu Y., Wang X., Liu L. (2017). Nosocomial and Community-Acquired Spontaneous Bacterial Peritonitis in patients with liver cirrhosis in China: Comparative Microbiology and Therapeutic Implications. Sci. Rep..

[54] Bert F., Andreu M., Durand F., Degos F., Galdbart J.O., Moreau R., Branger C., Lambert-Zechovsky N., Valla D. (2003). Nosocomial and community-acquired spontaneous bacterial peritonitis: Comparative microbiology and therapeutic implications. Eur. J. Clin. Microbiol. Infect. Dis..

[55] Ning N.Z., Li T., Zhang J.L., Qu F., Huang J., Liu X., Li Z., Geng W., Fu J.L., Huan W. (2018). Clinical and bacteriological features and prognosis of ascitic fluid infection in Chinese patients with cirrhosis. BMC Infect. Dis..

[56] Ding X., Yu Y., Chen M., Wang C., Kang Y., Lou J. (2019). Causative agents and outcome of spontaneous bacterial peritonitis in cirrhotic patients: Community-acquired versus nosocomial infections. BMC Infect. Dis..

[57] Popoiag R.E., Panaitescu E., Suceveanu A.I., Suceveanu A.P., Micu S.I., Mazilu L., Parepa I., Voinea F., Costea D.O., Enache F. (2021). Spontaneous bacterial peritonitis mortality trends of cirrhotic patients in the last decade in Constanta County. Exp. Ther. Med..

[58] Al-Ghamdi H., Al-Harbi N., Mokhtar H., Daffallah M., Memon Y., Aljumah A.A., Sanai F.M. (2019). Changes in the patterns and microbiology of spontaneous bacterial peritonitis: Analysis of 200 cirrhotic patients. Acta Gastroenterol. Belg..

[59] Cazacu S.M., Alexandru D.O., Popescu A.V., Popa P., Rogoveanu I., Iovanescu V.F. (2025). Is There a Role for the Neutrophil-to-Lymphocyte Ratio for Rebleeding and Mortality Risk Prediction in Acute Variceal Bleeding? A Comparative 5-Year Retrospective Study. Diseases.

[60] Metawea M.I., Moteleub H.N.A.E. (2022). Diagnostic role of simple indices in HCV-related liver cirrhosis outcomes: A prospective cross-sectional study. Clin. Exp. Hepatol..

[61] Wang X., Feng H., Hui Y., Yu Z., Zhao T., Mao L., Lin L., Wang B., Fan X., Yu Q. (2022). Neutrophil-to-lymphocyte ratio is associated with malnutrition risk estimated by the Royal Free Hospital-Nutritional Prioritizing Tool in hospitalized cirrhosis. JPEN J. Parenter. Enter. Nutr..

[62] Peng J., Chen H., Chen Z., Tan J., Wu F., Li X. (2025). Prognostic value of neutrophil-to-lymphocyte ratio in patients with hepatocellular carcinoma receiving curative therapies: A systematic review and meta-analysis. BMC Cancer.

[63] Qi X., Li J., Deng H., Li H., Su C., Guo X. (2016). Neutrophil-to-lymphocyte ratio for the prognostic assessment of hepatocellular carcinoma: A systematic review and meta-analysis of observational studies. Oncotarget.

